# Moving the mean with feedback: insights from behavioural science

**DOI:** 10.1038/npjpcrm.2016.18

**Published:** 2016-04-28

**Authors:** Jeffrey A Linder

**Affiliations:** 1 Brigham and Women’s Hospital, Division of General Internal Medicine and Primary Care, Harvard Medical School, Boston, MA, USA

Giving effective feedback is hard. In *Thinking Fast and Slow*, Daniel Kahneman^1^ describes a time when he was giving a lecture to Israeli fighter pilots about effective training practices. During his talk, Kahneman discussed the well-supported concept that rewarding good performance is more effective than punishing poor performance. After the talk, an incredulous senior instructor confronted Kahneman and said that criticising his trainee pilots for poor execution of aerial manoeuvers worked. The instructor had noticed that when he criticised trainees after they poorly executed a manoeuver, they almost always improved on their next attempt.

Kahneman saw a teaching opportunity. He drew a chalk target on the floor, had the officers turn their backs to the target and try to hit the target with two successive no-look coin throws. Those familiar with regression to the mean can guess what happened: officers who were far from the target on their first throw generally improved on the second throw; officers who were close to the target on their first throw generally did worse on their second throw. Kahneman showed how easy it is to conflate ‘effective feedback’—either positive or negative—with regression to the mean.

Designers of feedback interventions frequently make related mistakes. One classic error is to show people, without additional context, where they are on a bell curve. For example, my primary care group received feedback about our ordering of high-cost imaging for low-back pain. Although such feedback might change the behaviour of doctors who order too many high-cost imaging tests, it also can result in a ‘boomerang effect’ among doctors who order very few.^2^ Low utilisers see they are ordering fewer tests than their colleagues and give themselves licence to increase their ordering. This may be part of the reason that feedback has not been shown to be effective in reducing imaging for low-back pain.^3^ Variability decreases—poor performers improve and top performers worsen—but the mean does not move.

In the linked article, Vervloet *et al.*^4^ describe an intervention that included feedback to discourage antibiotic prescribing for acute respiratory tract infections in North-Limburg, Netherlands. The investigators matched and randomised eight family physician/pharmacist collaborative groups. The intervention consisted of advice in the family physicians’ electronic prescribing system, communication skills training and, 3 months after the communication skills training, a one-time feedback session about the group’s antibiotic prescribing rate.

Antibiotic prescribing in both the control and intervention groups decreased, and there was no overall effect of the intervention (although the intervention was effective among adolescent and adult patients). Several challenges may explain the negative overall trial result. There appeared to be a strong Hawthorne effect among the intervention groups and 2 of the 4 control groups. The authors examined their result in a simple pre-post analysis. A more sophisticated interrupted time series analysis might have detected changes in the trajectory of the antibiotic prescribing rate. In addition, the feedback intervention was particularly weak.

How could the feedback intervention have been improved? First, the feedback was only delivered once. To be effective, feedback should be delivered frequently. Second, the feedback was delivered at the group level. As the investigators noted, 81% of the variability in antibiotic prescribing was at the individual physician level. To be effective, feedback should be individualised and have the target of the feedback be under the recipient’s control. Third, the investigators do not describe exactly how the feedback was delivered, but such details are crucial to understanding additional reasons for which the intervention might not have been effective.^5^

My colleagues and I recently reported the results of a cluster randomised trial in 47 primary care practices in the United States of three behavioural interventions to decrease antibiotic prescribing for acute respiratory tract infections.^6^ We too saw a large Hawthorne effect in the control practices, with antibiotic prescribing rates for non-antibiotic-appropriate diagnoses—nonspecific upper respiratory tract infections, acute bronchitis and influenza—decreasing from 24 to 13%. Our feedback intervention, which we called ‘peer comparison,’ was significantly better. Peer comparison decreased inappropriate antibiotic prescribing from 20 to 4%.

The peer comparison intervention had several features that helped with effectiveness. Physicians received individual feedback about their own prescribing regularly, every month for 18 months. The feedback was for a highly specific subset of patients about whom we were nearly certain that antibiotics were inappropriate. We used a high-performing referent to which we provided positive reinforcement. We told the top performing doctors—those with the lowest antibiotic prescribing rate—just that: ‘You are a Top Performer.’ We gave the doctors with higher antibiotic prescribing rates the message that ‘You are not a Top Performer.’ Such emotionally laden feedback is attention-getting and connotes social approval or disapproval that can prevent boomerang effects.^2^

In another recent, large study of social norm feedback, Hallsworth *et al.*^7^ randomly assigned 1,500 high-antibiotic-prescribing general practices in England to receive a letter from England’s Chief Medical Officer. There was a statistically significant difference in antibiotic prescribing between control practices (131 antibiotic prescriptions per 1000 population) and intervention practices (127 antibiotic prescriptions per 1000 population) in the following 6 months. The effect size was small, but the intervention was simple and the reach was very broad. The investigators attributed the effectiveness of the intervention to comparison with local social norms, the high-profile nature of the feedback-giver, and presenting three specific, feasible actions recipients could undertake (providing self-care advice, offering delayed antibiotic prescriptions and discussing the feedback with colleagues).

Beyond feedback, we and others have responded to recent calls to use behavioural science to examine or improve antibiotic prescribing.^8^ In a retrospective study, we examined a contextual factor, time-of-day, to show that clinicians may be in a different cognitive state and are more likely to prescribe antibiotics later in their clinical sessions, when they might have greater ‘decision fatigue.’^9^ In a survey-based study of framing effects, we found that physicians were 12% more likely to prescribe aggressive treatments, such as broader-spectrum antibiotics, when aggressive treatments were presented individually rather than as part of a group.^10^ In a randomised trial, we demonstrated the effectiveness of signed, personalised, precommitment posters to reduce antibiotic prescribing.^11^

Most recently, we published a preliminary analysis examining the association between antibiotic prescribing and ‘cognitive reflection.’^12^ Cognitive reflection refers to the tendency of some people to make snap decisions without effortful thought and others’ tendency to make more considered decisions. In fact, cognitive reflection is related to the title and main point of Kahnemann’s book, *Thinking Fast and Slow.* We hypothesised that clinicians who tended to ‘think fast’ would make snap decisions and would have higher antibiotic prescribing rates. We also thought that clinicians who tended to ‘think slow’ would expend the effort to consider why antibiotics were not necessary and have lower antibiotic prescribing rates. However, we were surprised to find that there appeared to be a ‘sweet spot’ of cognitive reflection above and below which clinicians had a higher antibiotic prescribing rate. The effect size was small, about a 5% absolute difference in antibiotic prescribing, but clearly we or others need to do more work to better understand the relationship between cognitive reflection and prescribing.

Behavioural science has revealed many tools to enable us to look beyond regression to the mean and, instead, move the mean on appropriate prescribing. The Hawthorne effect needs to be anticipated and accounted for, but it is powerful and is underused as an intervention in its own right. Clinicians’ different cognitive states and cognitive styles may be related to prescribing and may modify the effectiveness of different interventions. Finally, feedback can be effective, but it needs to be well designed. Feedback should be regular, frequent, actionable, directed at people who have control over making change, employ carefully chosen comparators, and include a sense of social approval to move the mean in the desired direction.

## Funding

J.A.L. has received grant funding from Astellas, Pharma Inc. on an unrelated topic.
